# Novel *PTRF* mutation in a child with mild myopathy and very mild congenital lipodystrophy

**DOI:** 10.1186/1471-2350-14-89

**Published:** 2013-09-11

**Authors:** Anna Ardissone, Cinzia Bragato, Lorella Caffi, Flavia Blasevich, Sabrina Maestrini, Maria Luisa Bianchi, Lucia Morandi, Isabella Moroni, Marina Mora

**Affiliations:** 1 Child Neurology Department, Fondazione IRCCS Istituto Neurologico C. Besta, Milano, Italy; 2 Neuromuscular Diseases and Neuroimmunology Unit, Fondazione IRCCS Istituto Neurologico C. Besta, Milano, Italy; 3 Child Neurology Unit, Ospedali Riuniti di Bergamo, Milano, Italy; 4 Molecular Biology Laboratory, Istituto Auxologico Italiano IRCCS, Verbania, Italy; 5 Bone Metabolism Unit, Istituto Auxologico Italiano, IRCCS, Milano, Italy; 6 Muscle Cell Biology Lab, Fondazione IRCCS Istituto Neurologico “C. Besta”, Via Temolo 4, 20126, Milano, Italy

**Keywords:** Congenital generalized lipodystrophy type 4, Cavin-1, *PTRF/CAVIN*, Muscle mounding, HyperCKemia

## Abstract

**Background:**

Mutations in the *PTRF* gene, coding for cavin-1, cause congenital generalized lipodystrophy type 4 (CGL4) associated with myopathy. In CGL4, symptoms are variable comprising, in addition to myopathy, smooth and skeletal muscle hypertrophy, cardiac arrhythmias, and skeletal abnormalities. Secondary features are atlantoaxial instability, acanthosis nigricans, hepatomegaly, umbilical prominence and metabolic abnormalities related to insulin resistance, such as diabetes mellitus, hyperlipidemia and hepatic steatosis.

**Case presentation:**

We describe a 3 year-old child of Moroccan origin with mild muscle phenotype, mainly characterized by mounding, muscle pain, hyperCKemia and mild caveolin 3 reduction on muscle biopsy. No *CAV3* gene mutation was detected; instead we found a novel mutation, a homozygous single base pair deletion, in the *PTRF* gene. Only after detection of this mutation a mild generalized loss of subcutaneous fat, at first underestimated, was noticed and the diagnosis of lipodystrophy inferred.

**Conclusions:**

The *PTRF* gene should be investigated in patients with hyperCKemia, mild myopathy associated with spontaneous or percussion-induced muscle contractions like rippling or mounding, and no *CAV3* mutation. The analysis should be performed even if cardiac or metabolic alterations are absent, particularly in young patients in whom lipodystrophy may be difficult to ascertain.

## Background

Congenital generalized lipodystrophies (CGLs) are autosomal recessive disorders characterized by almost complete absence of body fat. Various gene defects may cause the condition. Mutations in a new gene, *PTRF (*polymerase I and transcript release factor), coding for cavin-1, have recently been found to cause CGL type 4 (CGL4) associated with muscle disease [1-3].

Cavin-1 is an abundant protein component of caveolae, and member of a family of related proteins that form a caveola- associated multiprotein complex. The complex also contains serum deprivation response (SDR)/cavin-2, SDR-related gene product that binds to C kinase (SRBC)/cavin-3, and muscle-restricted coiled-coil protein (MURC)/cavin-4 [4-8].

This complex can constitutively assemble in cytosol and associate with caveolin at plasma membrane caveolae. Cavin-1, but not other cavins, can induce caveola formation in a heterologous system and is required for recruitment of the cavin complex to caveolae [9]. Absence of cavin-1 leads to global loss of caveolae, and markedly diminished expression of all three caveolin protein isoforms, while caveolin mRNA levels are normal or above normal [10].

Cavin-1-knockout mice are viable and of normal weight but have high circulating triglycerides, low adipose tissue mass, glucose intolerance, and hyperinsulinemia (lipodystrophic phenotype).

Few *PTRF* mutations have so far been reported in humans [1-3,11,12]. We describe a 3-year-old boy with mild muscular phenotype in whom lipodystrophy was diagnosed only after detection of a novel homozygous mutation in the *PTRF* gene.

## Case presentation

The boy is the fourth child of healthy consanguineous (first cousins) parents of Moroccan origin. A sister and brother are in good health but the other sister died 6 hours after birth. Family history is negative for neuromuscular diseases.

The patient was born after an uncomplicated pregnancy with APGAR 8 at 1 minute and 9 at 5 minutes. He was noticed to have supinated left foot varus, mild dysmorphic features and reduced motility without hypotonia. Brain and renal ultrasound, EEG, and karyotype were normal. Normal psychomotor development was reported during the first year, but by 18–20 months he had clumsy gait, muscle pain in the lower limbs, mild fatigability and elevated muscle enzymes (CK 1179–1589 IU/L, normal 24–195; LDH 446–697 IU/L, normal 230–480); ECG and thyroid function tests were normal.

**Figure 1:**
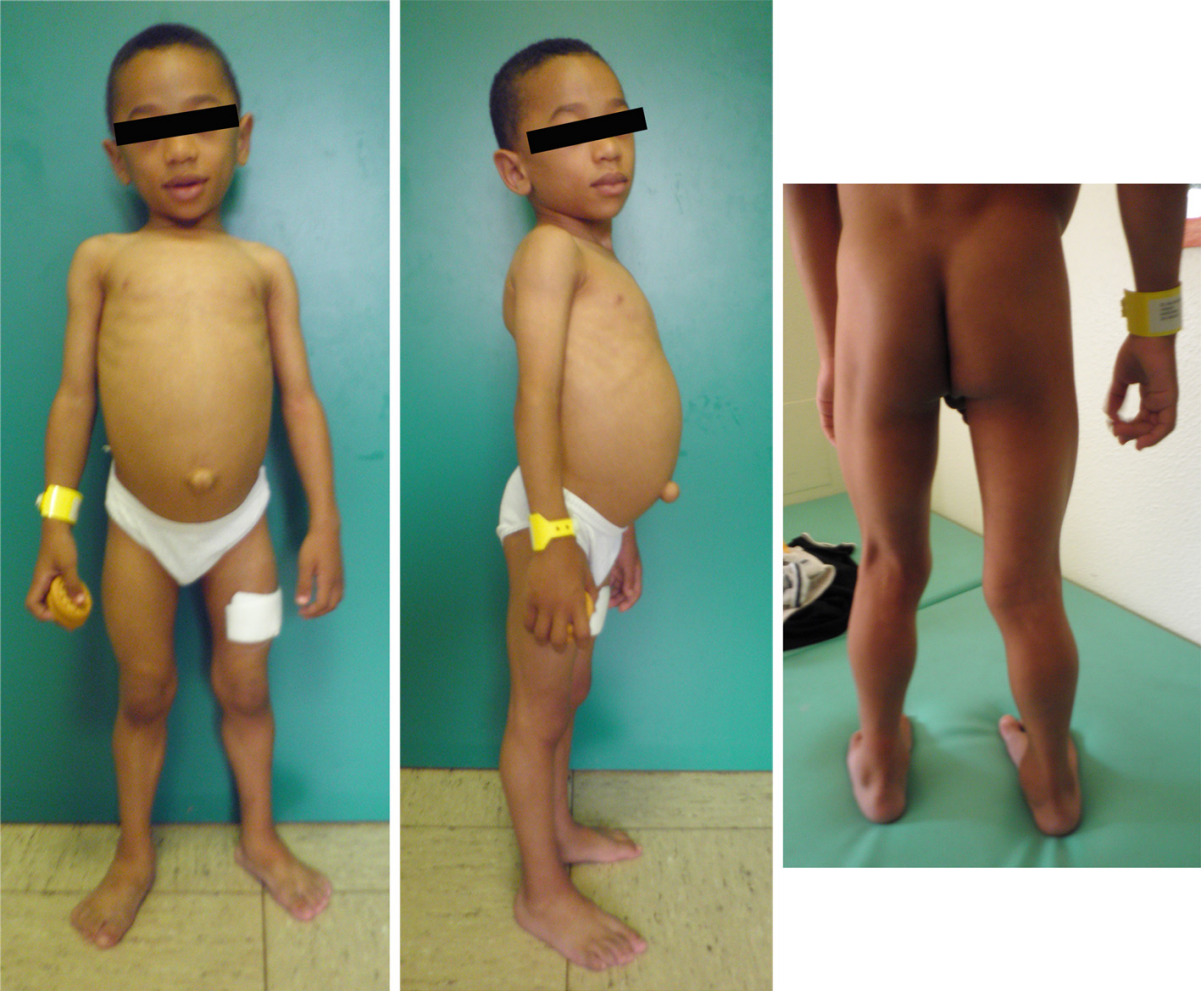
**Photographs of our patient.** Protruding abdomen, marked umbilical prominence, mild generalized loss of subcutaneous fat, and hypertrophic buttock and lower limb muscles are shown.

When next seen at 3 years 4 months, the boy had a protruding abdomen, a marked umbilical prominence, mild generalized loss of subcutaneous fat and no acanthosis nigricans (Figure 1); his weight was 13 kg (3rd to 10th centile), height 94 cm (50th centile). Neurological examination showed normal cranial nerves, normal tone, mild axial weakness, with difficulty in sitting from supine position, but normal strength limb muscles, hypertrophic buttock and lower limb muscles (Figure 1), reduced lower limb tendon reflexes, lumbar lordosis, supinated left foot varus and clumsy gait. The patient was able to get up from the chair and to stand up from the floor without support. There was also marked and rapid percussion-induced muscle contraction and mounding in upper and lower limbs.

Cognitive development was normal. Muscle enzymes were elevated: CK 963 (normal < 195 IU/L); LDH 767 (normal < 480 IU/L); AST 45 (normal < 41 IU/L); ALT 50 (normal < 37 IU/L); aldolase 19.2 IU/L (normal < 7.6).

**Figure 2:**
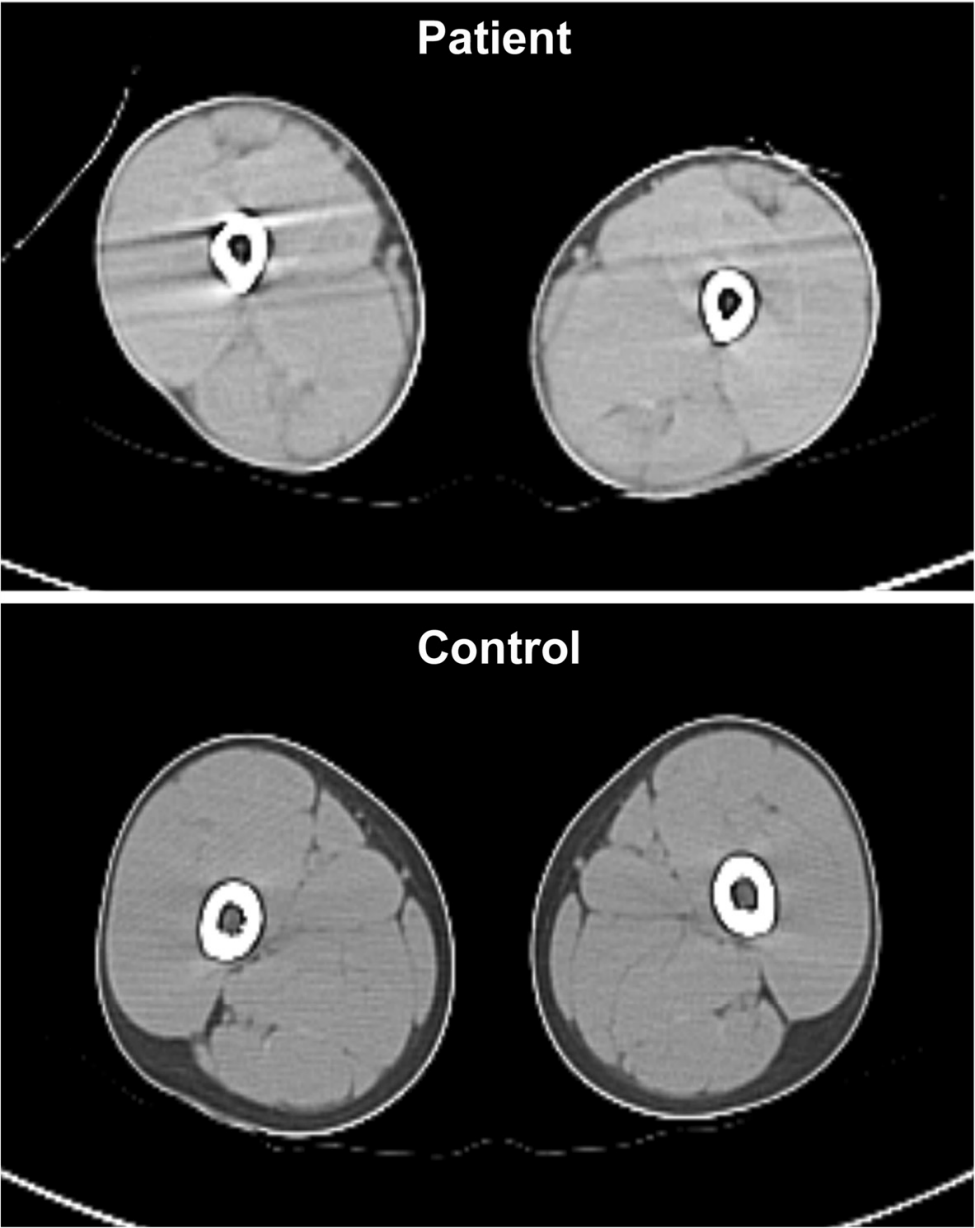
Lower limb CT, showing muscle hypertrophy and marked loss of subcutaneous fat.

Brain and cervical spinal cord MRI were normal; EMG revealed mild myopathic alterations and absence of myotonic discharges. Lower limb CT showed muscle hypertrophy and marked loss of subcutaneous fat (Figure 2). ECG was normal; abdominal ultrasonography was normal: in particular liver and spleen enlargements were excluded.

**Figure 3:**
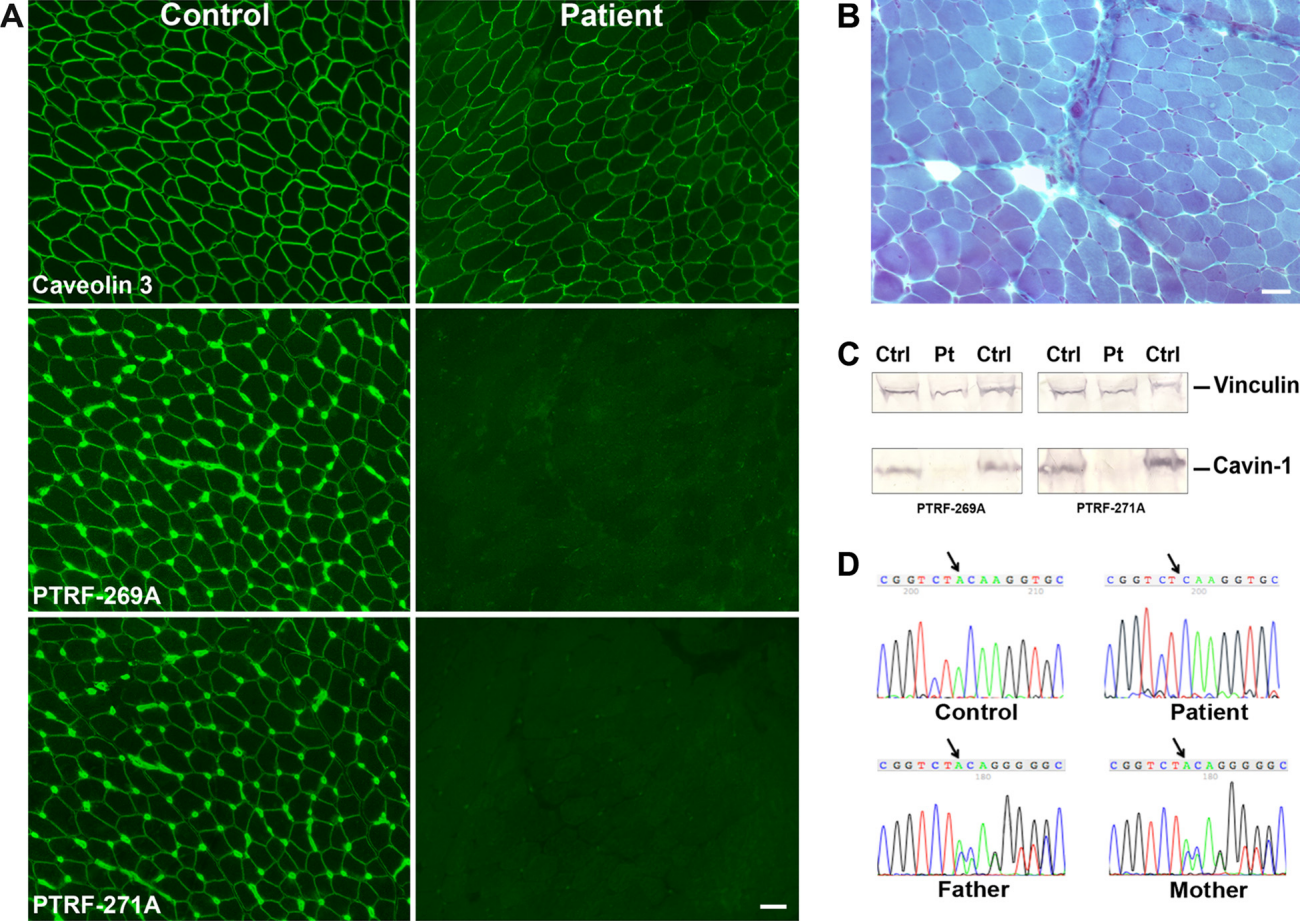
**Histological, immunochemical and molecular studies. (A)** Immunohistochemistry showing mild and irregular reduction of caveolin 3 and absence of cavin-1 (Bar = 20 μm); **(B)** Gomori trichrome staining showing mild histopathologic changes (Bar = 20 μm); **(C)** immunoblot showing absence of the band corresponding to cavin-1; **(D)** electropherograms showing homozygous single bp deletion in the patient, present in a single copy in both parents.

Muscle biopsy, after informed parental consent, showed moderate variation in fiber size, fibers with central nuclei, and mild endomysial fibrosis (Figure 3B). Muscle immunostaining showed mildly reduced and variable expression between fibers of caveolin-3 (Figure 3A), but *CAV3* gene sequencing failed to reveal any pathogenic mutation.

On sequencing the *PTRF* gene, a novel homozygous single base pair deletion, c.947delA was found (Figure 3D); the same mutation was present heterozygously in each parent and the healthy brother, and was absent in the healthy sister. The deletion was also detected in the cDNA and results in a frame shift and in introduction of the first stop codon 27 residues downstream of the correct stop codon, so the predicted protein is larger than the non-mutated protein (see ClustalW [13] predicted DNA sequence in Additional file 1: Figure S1).

Immunostaining showed that cavin-1 was absent from the sarcolemma with both antibodies used (Figure 3A). Western Blot showed absence of the band corresponding to cavin-1 (Figure 3C).

At the last visit, 14 months after diagnosis, clinical examination was unchanged with regard to the presence of generalized lypodistrophy; myopathic signs had remained mild, while fatigability and axial weakness were slightly improved and minimal acanthosis in the knees was observed.

After diagnosis the child underwent cardiological and metabolic evaluation. Echocardiogram was normal, ECG-holter monitoring showed sinus arrhythmia and few supraventricular extrasystoles, without evidence of QT prolongation. Fasting glucose, glucose tolerance test, growth hormone, cholesterol and triglyceride levels were normal. Serum leptin level was undetectable and adiponectin level was 0.83 μg/ml (normal 3–29 μg/ml). Finally, the patient had no history of recurrent pneumonia, and immunoglobulins were normal.

## Discussion

There are few reports of *PTRF* gene mutations in the literature [1-3,11,12]. They have been found in families of Omani, UK, Japanese, Hispanic and Turkish origin. We have found a new mutation in this gene in a mildly affected child of Moroccan origin. CGL4 was not diagnosed at first, and the *PTRF* gene was only investigated because muscle hypertrophy was associated with marked muscle mounding and CAV3 mutations were not found. After the mutation had been detected, the patient was re-evaluated and absence of adipose tissue noted, however the lipodystrophy in this patient was mild and subtle.

In contrast to “classic” CGL variants, symptoms are more variable in CGL4, comprising myopathy, smooth and skeletal muscle hypertrophy, cardiac arrhythmias, and skeletal abnormalities [3]. Secondary features are atlantoaxial instability, hepatomegaly, mild metabolic complications such as high circulating triglycerides and hyperinsulinism or diabetes, low leptin and adiponectin levels, reduction of growth hormone levels, immunoglobulin A deficiency, and umbilical prominence. Of these symptoms our patient had mild myopathic signs, mainly characterized by axial weakness, muscle hypertrophy, more evident in lower limbs, diffuse muscle mounding, umbilical prominence and low leptin and adiponectin levels. Other signs were absent. In particular, cardiac function was essentially normal; however the patient is still young and will require close monitoring as he grows, since the cardiac arrhythmias typical of *PTRF* mutated patients are potentially life-threatening: sudden deaths have been reported in consanguineous Omani families with *PTRF* mutations [14]. A case of similar age, but with more severe clinical features, has been recently described by Murakami et al. [12]. Differently from our patient, Murakami’s case had motor delay, more severe muscle impairment, progressive metabolic abnormalities and dystrophic changes on muscle biopsy.

Although the precise mechanism of muscle rippling is unclear, some reports indicate that caveolin-3 has a role in Ca^2+^ homeostasis. Loss of this protein leads to microscopic disarray in the colocalization of voltage-sensing dihydropyridine receptor and ryanodine receptor, thereby reducing the efficiency of excitation-contraction coupling [15].

As Cavin-1 is important in caveola formation and associates with caveolins, including caveolin-3, it is not surprising that mutations in cavin-1 cause muscle rippling or mounding.

The mutation we found affects the C terminus and creates a frame shift and a new stop codon 27 residues downstream of the original stop codon, producing a predicted protein of higher molecular weight. However, immunohistochemistry and Western blot indicates that the protein was absent from the patient’s muscle, probably because it is unstable and immediately degraded.

## Conclusions

To conclude, our 3-year-old boy with CGL4 had a mild muscular phenotype mainly characterized by mounding, muscle pain and hyperCKemia. We failed to detect a *CAV3* mutation, but instead found a novel homozygous mutation in *PTRF*. Our findings suggest that, in the presence of myopathy associated with spontaneous or percussion-induced muscle contractions (rippling or mounding), mildly reduced caveolin 3 expression and no *CAV3* mutation, the *PTRF* gene should be investigated, particularly in young patients in whom lipodystrophy may be difficult to ascertain.

## Consent

Parental written informed consent was obtained for publication of this case report and any accompanying images. A copy of the written consent is available for review by the Editor of this journal.

## Abbreviations

CGLs: Congenital generalized lipodystrophies; CT: Computed tomography; EEG: Electroencephalography; EMG: Electromyograohy; LDH: Lactate dehydrogenase; ALT: Alanine transaminase; AST: Aspartate transaminase; MRI: Magnetic resonance imaging; MURC: Muscle-restricted coiled-coil protein; PTRF: Polymerase I and transcript release factor; SDR: Serum deprivation response; SRBC: *SDR*-related gene product that binds to C kinase.

## Competing interests

The authors declare that they have no competing interests.

## Authors’ contributions

AA, CB, MM, IM conceived and designed the experiments and wrote the manuscript; CB carried out the molecular analysis; FB performed immunochemical evaluation of muscle biopsies and prepared the figures; SM and MLB performed biochemical studies; LM performed muscle biopsy and histological evaluation and critically revised the manuscript; AA, LC and IM performed clinical studies. All authors read and approved the final manuscript.

## Pre-publication history

The pre-publication history for this paper can be accessed here:

http://www.biomedcentral.com/1471-2350/14/89/prepub

## Supplementary Material

Additional file 1: Figure S1 Alignment of wild type and mutated PTRF gene using ClustalW2 (http://www.ebi.ac.uk/Tools/msa/clustalw2/). The deleted nucleotide is highlighted as well as the original stop codon and the new stop codon introduced by the mutation. Click here for file
