# Supplementary material for: Novel PTRF mutation in a child with mild myopathy and very mild congenital lipodystrophy
Source: BMC Med Genet. 2013 Sep 11;14:89. doi: 10.1186/1471-2350-14-89 (PMC3846852; doi:10.1186/1471-2350-14-89)
Supplement: Additional file 1: Figure S1 — Alignment of wild type and mutated PTRF gene using ClustalW2 (http://www.ebi.ac.uk/Tools/msa/clustalw2/). The deleted nucleotide is highlighted as well as the original stop codon and the new stop codon introduced by the mutation. [file 1471-2350-14-89-S1.pdf]

CLUSTAL 2.1 multiple sequence alignment

```
Original        ACCTGATTCCGTTCCTCAGCTCGCATTCCTCCGCCCGTCCCGCTCCCGCCGCCAGCGGAG 60
Mutated         ACCTGATTCCGTTCCTCAGCTCGCATTCCTCCGCCCGTCCCGCTCCCGCCGCCAGCGGAG 60
                ************************************************************

Original        GCCCTAGTCTCCCGCTCCAACTATTCCAACCATCCCGGGAAGGGTGGGGCGCTCGGCTCT 120
Mutated         GCCCTAGTCTCCCGCTCCAACTATTCCAACCATCCCGGGAAGGGTGGGGCGCTCGGCTCT 120
                ************************************************************

Original        TGGGTCCCCCTCCGCCGCCCCGCCTCGTCGATCTCCCCTTCTGCCCCGGTCCCTCCCTTT 180
Mutated         TGGGTCCCCCTCCGCCGCCCCGCCTCGTCGATCTCCCCTTCTGCCCCGGTCCCTCCCTTT 180
                ************************************************************

Original        CTGGGGTGGGGCCAGCCAATCAGCGATCAGACTCCGGAGTTTGGCCCGGGAGCTGGGGAG 240
Mutated         CTGGGGTGGGGCCAGCCAATCAGCGATCAGACTCCGGAGTTTGGCCCGGGAGCTGGGGAG 240
                ************************************************************

Original        CTCACCGATCCCCCGCCCAGCAGTTCTGGCCGCTGTCCCGGTGCGCACGGACGTGGCTCG 300
Mutated         CTCACCGATCCCCCGCCCAGCAGTTCTGGCCGCTGTCCCGGTGCGCACGGACGTGGCTCG 300
                ************************************************************

Original        AGTTTCCTCTGCTCTCCGCTCTCGCCCGCTAGCTCTCCTCCCTTCCGCTCCTGCTTCTCT 360
Mutated         AGTTTCCTCTGCTCTCCGCTCTCGCCCGCTAGCTCTCCTCCCTTCCGCTCCTGCTTCTCT 360
                ************************************************************

Original        CCGGGTCTCCCGCTCCAGCTCCAGCCCCACCCGGCCGGTCCCGCACGGCTCCGGGTAGCC 420
Mutated         CCGGGTCTCCCGCTCCAGCTCCAGCCCCACCCGGCCGGTCCCGCACGGCTCCGGGTAGCC 420
                ************************************************************

Original        ATGGAGGACCCCACGCTCTATATTGTCGAGCGGCCGCTTCCCGGGTACCCCGACGCCGAG 480
Mutated         ATGGAGGACCCCACGCTCTATATTGTCGAGCGGCCGCTTCCCGGGTACCCCGACGCCGAG 480
                ************************************************************

Original        GCCCCGGAGCCTTCCTCCGCTGGGGCTCAGGCAGCGGAGGAGCCGTCGGGGGCCGGCTCA 540
Mutated         GCCCCGGAGCCTTCCTCCGCTGGGGCTCAGGCAGCGGAGGAGCCGTCGGGGGCCGGCTCA 540
                ************************************************************

Original        GAAGAGCTGATCAAGTCGGACCAGGTGAACGGCGTGCTGGTGCTGAGCCTCCTGGACAAA 600
Mutated         GAAGAGCTGATCAAGTCGGACCAGGTGAACGGCGTGCTGGTGCTGAGCCTCCTGGACAAA 600
                ************************************************************

Original        ATCATCGGGGCCGTAGACCAGATCCAGCTGACTCAAGCACAGCTGGAGGAGCGGCAGGCG 660
Mutated         ATCATCGGGGCCGTAGACCAGATCCAGCTGACTCAAGCACAGCTGGAGGAGCGGCAGGCG 660
                ************************************************************

Original        GAGATGGAGGGCGCAGTGCAGAGCATCCAGGGCGAGCTGAGCAAGCTGGGCAAGGCGCAC 720
Mutated         GAGATGGAGGGCGCAGTGCAGAGCATCCAGGGCGAGCTGAGCAAGCTGGGCAAGGCGCAC 720
                ************************************************************

Original        GCCACCACGAGCAATACGGTGAGCAAGCTGCTGGAGAAGGTGCGCAAGGTCAGCGTCAAC 780
Mutated         GCCACCACGAGCAATACGGTGAGCAAGCTGCTGGAGAAGGTGCGCAAGGTCAGCGTCAAC 780
                ************************************************************

Original        GTGAAGACCGTGCGCGGCAGCCTGGAGCGCCAGGCGGGGCAGATCAAGAAGCTGGAGGTC 840
Mutated         GTGAAGACCGTGCGCGGCAGCCTGGAGCGCCAGGCGGGGCAGATCAAGAAGCTGGAGGTC 840
                ************************************************************

Original        AACGAGGCCGAGCTGCTGCGGCGCCGCAACTTTAAAGTCATGATCTACCAGGATGAAGTG 900
Mutated         AACGAGGCCGAGCTGCTGCGGCGCCGCAACTTTAAAGTCATGATCTACCAGGATGAAGTG 900
                ************************************************************
```

```
Original        AAGCTGCCGGCCAAACTGAGCATCAGCAAATCGCTGAAAGAGTCGGAGGCGCTGCCAGAG 960
Mutated         AAGCTGCCGGCCAAACTGAGCATCAGCAAATCGCTGAAAGAGTCGGAGGCGCTGCCAGAG 960
                ************************************************************

Original        AAGGAGGGCGAGGAGCTGGGCGAGGGCGAGCGGCCCGAGGAGGACGCAGCGGCGCTGGAG 1020
Mutated         AAGGAGGGCGAGGAGCTGGGCGAGGGCGAGCGGCCCGAGGAGGACGCAGCGGCGCTGGAG 1020
                ************************************************************

Original        CTTTCGTCGGACGAGGCGGTGGAGGTTGAGGAGGTTATTGAGGAGTCCCGCGCAGAGCGT 1080
Mutated         CTTTCGTCGGACGAGGCGGTGGAGGTTGAGGAGGTTATTGAGGAGTCCCGCGCAGAGCGT 1080
                ************************************************************

Original        ATCAAGCGCAGCGGCCTGCGGCGCGTGGACGACTTCAAGAAGGCCTTCTCCAAGGAGAAG 1140
Mutated         ATCAAGCGCAGCGGCCTGCGGCGCGTGGACGACTTCAAGAAGGCCTTCTCCAAGGAGAAG 1140
                ************************************************************

Original        ATGGAGAAGACCAAGGTGCGTACCCGCGAGAACCTGGAGAAGACGCGCCTCAAGACCAAG 1200
Mutated         ATGGAGAAGACCAAGGTGCGTACCCGCGAGAACCTGGAGAAGACGCGCCTCAAGACCAAG 1200
                ************************************************************

Original        GAAAACCTGGAGAAGACGCGGCACACCCTGGAGAAGCGCATGAACAAGCTGGGCACGCGC 1260
Mutated         GAAAACCTGGAGAAGACGCGGCACACCCTGGAGAAGCGCATGAACAAGCTGGGCACGCGC 1260
                ************************************************************

Original        CTGGTGCCCGCCGAGCGGCGCGAGAAACTGAAGACGTCGCGGGACAAGTTGCGCAAATCC 1320
Mutated         CTGGTGCCCGCCGAGCGGCGCGAGAAACTGAAGACGTCGCGGGACAAGTTGCGCAAATCC 1320
                ************************************************************

Original        TTCACGCCCGACCACGTGGTGTACGCGCGCTCCAAGACCGCGGTCTACAAGGTGCCACCC 1380
Mutated         TTCACGCCCGACCACGTGGTGTACGCGCGCTCCAAGACCGCGGTCT-CAAGGTGCCACCC 1379
                ********************************************** *************

Original        TTCACCTTCCACGTCAAGAAGATCCGCGAGGGCCAGGTGGAAGTGCTCAAGGCCACCGAG 1440
Mutated         TTCACCTTCCACGTCAAGAAGATCCGCGAGGGCCAGGTGGAAGTGCTCAAGGCCACCGAG 1439
                ************************************************************

Original        ATGGTGGAGGTGGGCGCCGACGACGACGAGGGCGGCGCGGAGCGCGGGGAGGCCGGCGAC 1500
Mutated         ATGGTGGAGGTGGGCGCCGACGACGACGAGGGCGGCGCGGAGCGCGGGGAGGCCGGCGAC 1499
                ************************************************************

Original        CTGCGGCGCGGGAGCAGCCCCGACGTGCACGCGCTGCTGGAGATCACCGAGGAGTCGGAC 1560
Mutated         CTGCGGCGCGGGAGCAGCCCCGACGTGCACGCGCTGCTGGAGATCACCGAGGAGTCGGAC 1559
                ************************************************************

Original        GCCGTGCTGGTGGACAAGAGCGACAGCGACTGA--------------------------- 1593
Mutated         GCCGTGCTGGTGGACAAGAGCGACAGCGACTGAGCCGCCCCCGCTGCCACCCACCCCATT 1619
                *********************************

Original        -------------------------------------------------------
Mutated         CCTCGCTCCTTCCGAACTTCCTCTTTCGCATTCTCTCTCGGCTCGAGCTGGCTGA 1674
```
